# Transmasculine Persons' Experiences of Encounters with Health Care Professionals Within Reproductive, Perinatal, and Sexual Health in Sweden: A Qualitative Interview Study

**DOI:** 10.1089/trgh.2020.0081

**Published:** 2021-12-02

**Authors:** Kristin Asklöv, Regina Ekenger, Carina Berterö

**Affiliations:** ^1^ Department of Obstetrics and Gynecology and Linköping University, Linköping, Sweden.; ^2^ Department of Biomedical and Clinical Sciences, Linköping University, Linköping, Sweden.; ^3^ Division of Nursing Science and Reproductive Health, Department of Health, Medicine, and Caring Sciences, Linköping University, Linköping, Sweden.

**Keywords:** encounter, experiences, reproductive and sexual health care, transgender, transmasculine

## Abstract

**Purpose:** Transmasculine persons may experience stigma, which increases the risk of psychological distress. They may need reproductive, perinatal, and sexual health care; however, qualitative studies addressing transgender individuals' experiences are scarce. This study aimed at interpreting and describing the experiences of transmasculine persons in encounters with health care professionals (HCPs) within reproductive, perinatal, and sexual health care.

**Methods:** Nine qualitative semi-structured online interviews were conducted via email with transmasculine persons, and data were analyzed by using Braun and Clarke's thematic analysis.

**Results:** Two themes were identified. The first theme is *normalization and confirmation of the gender identity.* This theme comprises the knowledge and experience that these transmasculine persons are facing. The verbal approach from the HCPs was important as well as could be addressed with a non-binary approach. The second theme is *Respect in an especially exposed situation.* This theme shows the great importance of being involved in the care and at the same time being met with openness and empathy. There must be good prospects of being able to preserve dignity.

**Conclusion:** Transmasculine persons are in an exposed position in reproductive, perinatal, and sexual health care. The encounters in health care could be negatively affected if HCPs show inadequate knowledge or express gender stereotypical attitudes. A good encounter is characterized by respect, preserved integrity, involvement in the care, and an open attitude toward gender variations.

## Introduction

The concept of gender is used to separate the biological and social sex,^1^ but it is also a tool for studying how people shape themselves and how this affects people's living conditions.^1^ A person's gender identity is described as the internal perception of a person's gender and how they are labeling themselves. Transgender is an umbrella term that includes gender-variant identities, not identifying with the sex assigned to them at birth or not fitting into the binary gender categories, either female or male or both.^2^

Transgender persons may experience isolation and stigma, which increases the risk of anxiety and depression.^3,4^ They also face barriers to health care, since it is common that transgender persons hesitate to seek health care due to fear of being mistreated or of facing ignorance by health care professionals (HCPs) if they should disclose their gender identity.^5^ Meanwhile, HCPs state that they require increased knowledge in the specific health care needs of transgender persons.^6^ Being recognized and addressed as the gender that they identify themselves as is an important issue for transgender persons,^2^ and they often experience the need to educate their HCPs and feel frustrated at having to do so.^7,8^ Sexual and reproductive rights specify that every person has the right to make decisions about their own body, and to be able to access health care to support their sexual and reproductive rights.^9^ However, there is a lack of knowledge within health care regarding transmasculine persons' sexual and reproductive health,^7,10^ and there is a need for increased information about fertility and family planning from transmasculine persons.^11^ A majority of transmasculine persons express a wish to become parents,^10,12^ and a quarter of them wish to become pregnant.^10^ Transmasculine persons who have experienced pregnancy have given evidence about different ways to experience and cope with their pregnancy.^13–15^ Many describe an increased gender dysphoria during the pregnancy,^14^ whereas others, on the contrary, experience a decreased gender dysphoria.^13,15^ Since many transmasculine persons choose to preserve their genitals, they are still in need of reproductive and sexual health care that traditionally is directed toward women. Transmasculine persons testify that it is important to receive gynecological health care but that the pelvic examination is a procedure that exposes them due to dysphoria-based discomfort^5,16^ and therefore they often end up avoiding gynecological health care.^7,17^ This avoidance could lead to an increased risk for cervical cancer among transgender men that remains undetected due to decreased screening.^16^

In Sweden, a cornerstone in public health work is to promote universal access, free of cost, to safe and secure sexuality and good reproductive health, thus also including more vulnerable individuals or groups. The midwifes have a central role in maternal health care in Sweden, which is unique in the world. Maternal health care works comprises the following areas of activity: health care in connection with pregnancy; support in parenting and parenting groups with childbirth and parental preparation; family planning at the individual level but also gynecological cell test control for preventing cervical cancer and preventing work regarding living habits. In Sweden, all women have access to reproductive, perinatal, and sexual health care at medical and gynecological clinics or youth guidance centers. The youth guidance centers are directed toward both young women and men. Transmasculine persons preserving their female genitals are included in this maternal health care work. The HCPs' knowledge about transgender is crucial due to its impact on whether the transmasculine persons avoid health care or not.^18^

## Methods

We focused on the transmasculine persons' experiences using an inductive qualitative approach,^19^ with experiential qualitative research, asking for lived experiences and perspectives.

Interviews were conducted via email, since that is appropriate when the area of research could be sensitive or stigmatizing,^20^ and it also made it possible to reach a larger geographical area.

### Sample and participants

Purposive sampling was used.^21^ Eligibility criteria were: to speak and understand Swedish, be older than 18 years, and to have been assigned female gender at birth. Recruitment was via 2 Facebook groups; a Swedish interest group on Facebook with about 600 members for people who were assigned female sex at birth but did not identify themselves as women and a Swedish interest group on Facebook concerned with queer questions, with about 350 members. An invitation to participate in the study was placed on these Facebook pages with the authors' contact details.^22^ The participants reported their interest in the study by using an already existing private email address of the researcher's. A welcome letter was sent out with information about the purpose of the study, the rights of the participants, and the approximate scope of the study. The letter ended with information that the applicants should respond to the existing private email address if they wanted to participate and that they could ask any questions about the study before the interview started.^23^ After the interviews, the participants were asked to recruit others and thus a snowball selection method was also used.

The study was performed in accordance with the Declaration of Helsinki and Swedish legislation of non-invasive studies.^24,25^ According to Swedish law, ethical approval is not required for research studies conducted during advanced educational programs, but all considerations in the study were made in accordance with ethical laws and guidelines. Participants were informed that they could leave the study at any time and the interview material would always remain confidential. Study participation was voluntary, and participants could withdraw at any time without restriction. Their response to the information letter was regarded as informed consent.^26^ Each participant was assigned a pseudonym to maintain confidentiality.

Several actions were taken to ensure data and participant confidentiality. The response feature was not used to minimize the amount of information in each email and thus ensure confidentiality.^27^ Some participants used email addresses with parts or all of their own names, so such email was handled with care^28^ and after the study was completed, the email address used to conduct the interviews was deleted. When the email text was exported to the analysis document, data were made anonymous.^22,27^

### Procedure

The interviews were conducted in January to February 2019. An interview guide was designed^19^ with one broad opening request: Please, tell us about your experiences of health care encounters. Follow-up questions were formulated on answers-narratives given, for example, Could you describe that situation? What happened then? The nature of the email interview was the same as in the traditional face-to-face interview, except that the interviewees wrote their answers.^22^ A pilot interview was conducted with a person who met our inclusion criteria to ensure that the questions we asked were answerable and that the answers corresponded to the purpose. This pilot interview was conducted via email exactly as the planned interviews.^29^ This pilot interview was later included in the results. No changes were made to the interview questions; however, the follow-up questions were developed. Data collection was performed via two to three email interviews at a time, which was experienced managable to not mixing given information.^23^ Two participants withdrew their participation after answering the two introductory questions. Two reminders were sent and since they did not reply to these, they were regarded as withdrawn. In total, 9 participants were interviewed by email, giving an amount of data consisting of 15,459 words.

### Analysis

Data were analyzed by using thematic analysis.^30^ Thematic Analysis is a flexible method that could be used both inductively or deductively, and semantic and/or latent. The method is not strictly connected to one perspective, and it has influences from several methodologies. In the first stage of the analysis, the material was read several times to ensure a deep understanding of the material was acquired and notes on potentially interesting aspects of the data were made at this stage. Second, the material was read through and data extracts corresponding to the purpose were picked out and coded, first by us individually, and then the extracted text and 562 codes were checked jointly. This coding was systematically working through the entire dataset, giving full and equal attention to each data item. Third, codes were grouped based on patterns in the data to form potential themes. This phase is on an abstraction level, moving up from the codes. Fourth, potential themes were critically examined in relation to the codes for each theme. The dataset was also critically examined in relation to the themes. Some codes were transferred to another theme or a new theme, and a thematic map was also created to give a visual overview of the eight themes developed. Relevant data were organized under each theme, and the dataset was read to confirm that the themes created had captured the participants' experiences and to verify that the themes had internal homogeneity and external heterogeneity. The map should show the content of the themes and how they are related. Fifth, the analysis proceeded by specifying each theme by writing short definitions and naming them. So, the themes were once again reduced since some themes were overlapping and there was more depth of the themes when they were fewer. It is better to have some few themes telling the story than a “bucket list” sorting the data. Finally, this analysis was written, quotes were extracted from the data extracts, and the result was produced. In the participants' citations, typos were corrected to facilitate reading and understanding of the data.^31^

## Results

### Sample characteristics

In total, nine transmasculine persons participated in the study, having wide heterogeneity regarding sexual orientation, such as asexual, queer, and heterosexual. They were between 25 and 43 years old (median age 33). They defined themselves as men, transgender, or transmasculine and all had been diagnosed with transsexualism or gender dysphoria. Six out of these nine persons had undergone mastectomy and out of these six persons, two had undergone genital surgery. Seven were treated with testosterone.

### Thematic result

During the analysis, we identified two themes answering the question how transmasculine persons experience encounters with HCPs within reproductive, perinatal, and sexual health. These themes were: Normalization and confirmation of the gender identity, and Respect in an especially exposed situation.

### Normalization and confirmation of the gender identity

When HCPs confirmed and normalized gender identity, transmasculine persons felt safer seeking and obtaining reproductive, perinatal, and sexual health care, whereas the opposite prevailed when caregivers pathologized and mistrusted their gender identity.

How HCPs used the language, a gender-neutral language, symbols and design of brochures, and correct referrals was important. The attitudes of HCPs and how care was designed based on binary or non-binary and normalizing or non-normalizing attitudes toward gender influenced whether they felt welcome and confirmed or excluded in care concerned with reproductive, perinatal, and sexual health. The HCPs could mix up gender identity and sexuality, which made the transmasculine persons feel distrusted regarding how they defined themselves. The participants were often met by astonishment and prying questions that were irrelevant to the care and as a result they felt strange and unusual.

“I hate when you get to certain places and they say ‘This is very unusual for us’ and start to ask a lot of questions (especially about bottom surgery). Like it is weird or that I would be different just because I was misfortunate enough to be born with body parts that don't belong and an incorrect hormone production.” Lucas, 25 years old.

The HCPs' knowledge and experience of transgender affected the participants' experience of the encounter. Ignorance forced the participants to educate their HCPs, which was perceived as exhausting. They felt safer if a clinic was certified within LGBTQ and if the HCPs had knowledge about gender and gender identities.

It was desirable, as a transmasculine person, to be asked about preferred pronoun and that the preferred pronoun was documented in the medical record. Questions about which words the participants wanted to use about their own bodies were desired. In conclusion, they felt confirmed in their gender identity when gender-neutral and inclusive language was used.

“She used words like ‘cisgender’, ‘cis male’ and ‘cis female’ which I find inclusive. When she talked about the ways the reproductive medicine clinic can help people to reproduce, she said, ‘the person with uterus/ovaries’, ‘the person who will carry the pregnancy’ and ‘person with penis/sperm’.” Liam, 33 years old.

The participants felt excluded because of the way the health care system is divided into female and male, binary health care. The fact that the clinic was often named a “women's clinic” made it uncomfortable and offensive and as a result they did not seek health care at all. The youth guidance center and the productive medicine clinic were described as gender-neutral clinics and therefore safer to visit, because they are directed toward both young women and men.

“If I had NOT been able to go to the youth guidance center and take my pap smear the alternative would have been not to take the pap smear at all, unfortunately. So, for that reason I do not know what will happen next time. Then I'll be too old for the youth guidance center and I don't know of any other place to do it that feels neutral.” Oscar, 26 years old.

### Respect in an especially exposed situation

The participants said that the encounters in this exposed situation had a great impact on how they experienced the health care within the reproductive, perinatal, and sexual area. These encounters caused strong emotions and they wanted to avoid them. A respectful encounter that included participation, the possibility to preserve one's integrity, and to be met with openness and empathy could make the situation more manageable.

Pelvic examinations were often introduced by the HCPs without preparation or explanation of why they needed to perform them. This could lead to feelings of powerlessness and the participants felt that they had to suck it up despite their anxieties. If they were informed and asked to consent to the examination, and told they could stop it whenever they wanted, they would feel respected in this situation.

“After our conversation she asked me abruptly to take off my pants because they needed to do an ultrasound on my ovaries and uterus. Next, I was sat on an exam table with a probe inserted inside of me and poking around. I remember that I almost cried. Maybe I was naive, but I had not been informed that there would be a pelvic exam.” William, 27 years old.

The participants describe it as nerve-racking to expose their bodies during a pelvic exam and said that it was hard to preserve their gender identity. This exposed situation made the transmasculine persons avoid seeking that kind of health care. Facilitating factors for preserving their integrity could be that the exam table was well placed in the room and that no medical students were present. An experienced HCP conducting a quick exam was positive since it shortened the time of exposure. The possibility to use a self-test to avoid the pelvic exam was a way to preserve their integrity.

Facilitating factors within the encounters included the HCPs having an open approach and a willingness to learn, admitting their own and the health care system's flaws. When the HCPs showed understanding of the transmasculine persons' situation and adjusted the care based on their needs it was described as an empathic approach, leading to strong positive experiences. Cancelling the examination and thereby acknowledging that the situation was hard for the participant was described as a sign of empathy. The empathic encounter made them feel seen and, consequently, respected.

“The fact that she was calm and knew that it was my first pelvic exam meant a lot, I think. She explained and was careful. Even though the tears fell, and she said comforting words I was stressed out.” Elias, 37 years old.

The opposite was described when HCPs expressed themselves in a way that felt discriminatory. That made the already exposed situation feel even worse.

## Discussion

The current study showed that the transmasculine persons sometimes felt excluded, questioned, and distrusted by HCPs about how they defined themselves. In contrast, at other times they felt seen and confirmed in their gender identity. An overall problem in this regard is that the health care system is built on a binary gender model where transgender persons are excluded. The informants could be hesitant to seek care in clinics that defined their patients, and which described themselves as “women's clinics.” Alternatively, the informants might hide their gender identity while seeking health care. Hiding one's gender identity as transgender because of a lack of cultural acceptance is common globally and can lead to inadequate health care.^2^ Previous studies show that transgender persons experience stigmatization by HCPs, which leads to avoidance of seeking health care.^32,33^ By minimizing transgender persons' exposure to stigma and prejudice there can be reduced stress and an increase of mental health for this population.^34^ The current study showed that the implementation of more gender-neutral HCPs might be a way to include transgender persons.

Ignorance and poor knowledge regarding transgender matters was regularly found in the current study. The participants' sex, gender, and sexuality were often confused, and they often had to educate their HCP, something that felt exhausting. Previous studies also describe the frustration and exhaustion that comes from having to educate one's HCP.^7,8^ The poor knowledge and low competence regarding transgender persons and their specific health care needs are coherently described by previous research.^6,7,35–38^ Offensive and degrading attitudes from HCPs were also found to be an issue in a previous study.^6^ In the current study, the experience of offensive comments and misuse of power were described. The participants wanted their situation to be considered normal and wished that the HCP would not emphasize that they were transgender. A normalization of transgender people and a focus on what is relevant to their medical care has been proven desirable.^8^ An ignorant approach had the consequence that the informants avoided seeking care. Previous studies have also shown that transmasculine persons avoid screening for sexual transmitted infections because of the HCPs' lack of cultural competence and poor knowledge about transmasculine persons and their specific sexual health care needs.^7,16^ In the current study, the participants thought that poor knowledge could be weighed up if the HCP showed openness and a willingness to learn and understand.

The findings from the present study showed that the HCPs' use of language and their preferred pronouns were significant, and the use of the right preferred pronoun gave a sense of feeling respected and cared for. As a result, the transmasculine person felt confirmed in their gender identity. Previous research provided evidence that the HCPs' incorrect use of pronouns creates a barrier between transmasculine persons and HCPs.^5,13,39^ It is also perceived as respectful when HCPs ask what name the patient prefers, to use the surname^8^ and to use gender-neutral language,^40^ which is also strengthened by the current study.

The pelvic examination was highlighted in the current study as something difficult to undergo. The consequence might be that the examinations are avoided.^41^ A previous study showed that transmasculine persons are of the opinion that it is important to have pelvic examinations despite their emotional conflict.^5^ How well the participants in the current study handled the pelvic exam depended on whether they were involved and well informed, whether they could preserve their integrity, and the HCPs' empathic approach. An empathic approach has been proven to increase the chance of the transgender person seeking health care again.^42^ The current study shows that lack of knowledge and competence regarding transmasculine persons and their specific health care needs are common and therefore there is a need for increased training and education. A recommendation for HCP education could be to follow the Transgender Care and Treatment Guideline,^43^ whereas both physiological, emotionally, and pelvic examination aspects are presented. When HCPs are educated and have knowledge about transgender issues, the transmasculine person will be confirmed in their gender identity, which will result in less hesitation to seek reproductive and sexual health care in the future. The research regarding transmasculine persons' experiences in obstetric care and contraceptive consultations is scant, and further research is needed for modern health care.

### Limitations

This study attempted to capture the experiences of transmasculine persons in encounters with HCPs in reproductive, perinatal, and sexual health care. This is a “hard-to-reach” population, so we used purposeful sampling with a modified snowball sampling approach.^19,21^ Using Facebook as a foundation for the “snowball sampling” facilitated the accessibility to these transmasculine persons. The participants volunteered to be a part of the study via other transmasculine persons, which may have introduced sampling bias. However, we wanted to reach these persons with these unique experiences, which outweigh the bias concern. Using Facebook could be thought to exclude persons not having access to the internet. In Sweden, ∼98% of the population have access to the Internet and use it. Using Facebook to communicate with other people in a similar situation and with similar experiences is common. Using Facebook as the foundation made it possible to reach participants all over the country, with no geographical limitations Finally, the sample size of nine participants might seem small, but rich and nuanced information was reached, which in qualitative research is the key point.^19.21^ The analysis was performed by nurses and midwives working with sexuality and reproductive health, including LGBTQ, and also experienced in qualitative research. During the whole analysis process, there were discussions among the researchers. Each stage of thematic analysis represents a reflexive process over time, and the analysis is moving backward and forward continuously, which may strengthen trustworthiness.^44^

## Abbreviation Used

HCP: health care professional
